# Identification and Characterization of a Cellodextrin Transporter in *Aspergillus niger*

**DOI:** 10.3389/fmicb.2020.00145

**Published:** 2020-02-07

**Authors:** Hui Lin, Jun Zhao, Qingqing Zhang, Shixiu Cui, Zhiliang Fan, Hongge Chen, Chaoguang Tian

**Affiliations:** ^1^College of Life Sciences, Henan Agricultural University, Zhengzhou, China; ^2^Department of Biological and Agricultural Engineering, University of California, Davis, Davis, CA, United States; ^3^Tianjin Institute of Industrial Biotechnology, Chinese Academy of Sciences, Tianjin, China

**Keywords:** *Aspergillus niger*, cellulose, cellodextrin transporter, cellobiose, cellodextrin

## Abstract

*Aspergillus niger* produces a wide spectrum of extracellular polysaccharide hydrolases that hydrolyze cellulose into soluble glucose and cellodextrins. Transporters are essential for sugar uptake, yet it is not clear whether cellodextrin transporters exist in *A. niger*. Here, one cellulose inducible cellodextrin transporter CtA was identified in *A. niger* B2. It was found that CtA not only could transport cellobiose, but also cellotriose, cellotetraose, and cellopentaose. The yeast strain YPβG-CtA, expressing cellodextrin transporter CtA and an intracellular β-glucosidase, grew on cellobiose with the cell growth rate of 0.0830 ± 0.0113 h^–1^ under aerobic condition. Furthermore, the engineered yeast could produce 1.1 g/L ethanol anaerobically on cellobiose in 2 days. The identification of CtA provides evidence that the cellodextrin uptake is a complementary strategy of cellulose utilization in *A. niger*, and the CtA could be a strong transporter candidate for constructing engineered cellodextrin-utilizing microorganisms.

## Introduction

The filamentous ascomycete fungus *Aspergillus niger* is ubiquitous in the environment and has been widely used in industry for the production of organic acids and extracellular enzymes (Okada, 1988). *A. niger* produces a wide spectrum of polysaccharide hydrolases, which enable it to grow on cellulosic biomass. The secreted cellulases hydrolyze cellulose into glucose and cellodextrins. The resulting sugars are then transported into the cells and get metabolized intracellularly. Characterization of the sugar transporters is therefore important for understanding the process of fungal plant biomass degradation.

Cellodextrin transporters from fungi commonly belong to the MFS (major facilitator superfamily) transporters, which are single polypeptide facilitating the movement of small soluble substrates in response to ion gradients (Yan, 2013). In the past years, several cellodextrin transporters have been identified in cellulolytic fungi. Two cellodextrin transporters (CDT-1 and CDT-2) were identified in cellulolytic fungus *Neurospora crassa* (Galazka et al., 2010), and three genes, *CdtC*, *CdtD*, and *CdtG*, encoding cellodextrin transporters have been reported in cellulolytic fungus *Penicillium oxalicum* (Li et al., 2013), and the transporter Stp1 and CltA involving cellobiose uptake were identified in *Trichoderma reesei* and *Aspergillus nidulans*, respectively (Zhang et al., 2013; dos Reis et al., 2016).

The genomic and transcriptomic analysis of *A. niger* showed that it contains 86 putative sugar transporters, which were classified as members of the Pfam family of “Sugar and other transporters” (PF00083) (Pel et al., 2007; de Vries et al., 2017; Peng et al., 2018). Until now, the high-affinity and low-affinity glucose transporters in *A. niger* have been identified and studied extensively (Torres et al., 1996; Vankuyk et al., 2004; Sloothaak et al., 2015), while the cellodextrin transporters in *A. niger* have not yet been identified. Recently, the abilities of transportation of 12 different sugars by 43 putative sugar transporters from *A. niger* were extensively analyzed by de Vries et al. (2017), yet these sugar transporters were not characterized for their cellodextrin transporter function. Several sugar transporters in *A. niger* were shown to be inducible by cellulosic biomass. Delmas et al. (2012) reported that 3 sugar transporter genes were up-regulated when *A. niger* grew on wheat straw and de Souza et al. (2011) found that 5 sugar transporter genes in *A. niger* were up-regulated on steam-exploded sugarcane bagasse, which indicate *A. niger* may have cellodextrin transporters.

The aim of this study was to identify and characterize the cellodextrin transporters in *A. niger*, to provide insight into the cellulose utilization mechanism of *A. niger*, and to provide potentially useful cellodextrin transporter for constructing efficient biofuel producer strains.

## Materials and Methods

### Strains, Media, and Culture Methods

*Aspergillus niger* B2 was isolated from rotting wheat straw in our laboratory (Yue et al., 2012), and it has been deposited in the China General Microbiological Culture Collection Center (CGMCC) with deposit number CGMCC No. 17078. Sugarcane bagasse was pretreated by steam explosion at 2.0 MPa for 150 s, and the steam-exploded sugarcane bagasse consists of 49 ± 0.3% cellulose, 23 ± 0.2% hemicellulose, 23 ± 0.2% lignin, and 5 ± 0.1% ash, which was determined by the NREL procedure: determination of structural carbohydrates and lignin in biomass (Sluiter et al., 2008). All the experiments were performed in technical and biological triplicates, and standard deviations of triplicates are represented by error bars. Cellobiose was purchased from Sigma-Aldrich (United States), cellotetraose was purchased from Elicityl (France), cellotriose and cellopentaose were purchased from TCI (Japan), and no glucose was detected in all the samples by HPLC (Supplementary Figure S1). All reagents were of analytical grade and were used without further purification, except where stated.

*Aspergillus niger* B2 was cultured on potato dextrose agar (PDA) solid medium 5 days for spores preparation. The modified Mandel’s medium for fungal spores germination and hyphal growth (growth media) contained glucose (20 g/L) and peptone (1 g/L) (Mandels and Weber, 1969). The modified Mandel’s media for inducing the transcription of cellodextrin transporters (induction media) contained steam-exploded sugarcane bagasse (5 g/L) and peptone (1 g/L).

*Saccharomyces cerevisiae* YPH499 strain was grown in synthetic media (SM) contained yeast nitrogen base without amino acids and ammonium sulfate (6.7 g/L), adenine 50 mg/L, histidine (20 mg/L), lysine (30 mg/L), tryptophan (20 mg/L), leucine (100 mg/L), uracil (20 mg/L), and glucose (20 g/L) or cellodextrins (5 g/L) as the sole carbon source at 30°C and 250 rpm. *S. cerevisiae* YPH499 harboring plasmid pRS425 was grown in the SM without leucine (SM-Leu), and *S. cerevisiae* YPH499 harboring plasmid pRS425 and plasmid pRS426 was grown in the SM without leucine and uracil (SM-Leu-Ura).

### RNA Extraction and Expression Profiling by RT-qPCR

To characterize the expression of potential cellodextrin transporters (*An12g09270*, *An14g01600*, *An13g03250*, *An03g05320*, *An08g09350*, *An16g06220*, and *An04g02790*) upon the induction of cellulosic biomass, 50 mL Mandel’s growth medium was inoculated with fresh *A. niger* B2 spore suspension in Mandel’s salt solution (about 10^7^ spores) and cultured at 200 rpm and 30°C for 48 h. Mycelia were harvested by vacuum filtration and washed with sterile Mandel’s salt solution three times, and then 1.5 g mycelia were transferred to 50 mL Mandel’s induction medium containing 0.5% of steam-exploded sugarcane bagasse, grown at 30°C and 200 rpm. Mycelia were harvested at 6 h, frozen in liquid nitrogen, stored in a freezer at −80°C, and then used for RNA isolation by using RNeasy Plant Mini Kit (Qiagen, Hilden, Germany, Cat. No. 74903) according to the manufacturer’s protocols.

The extracted mRNA samples were quantified on the NanoDrop^®^ 1000 (Thermo Fisher Scientific). mRNA was then reversed transcribed to cDNA by using the ProtoScript^®^ II Reverse Transcriptase (NEB), following the manufacturer’s menus. The primers used in RT-qPCR were listed in Supplementary Table S1. RT-qPCR reactions were performed using the Bio-Rad iQ5 Real-Time PCR System and the SYBR Green PCR Master Mix kit (Thermo Fisher Scientific, United States), following the manufacturer’s instructions. The reaction mixture containing 1.5 μL of cDNA (75 ng), 0.32 μL of primers mixture (100 μM forward primer and 100 μM reverse primer, 10 μM SYBR Green PCR Master Mix kit, and nuclease-free water up to 20 μL were dispensed into triplicate wells on a 384-well plate. The RT-qPCR protocol was initiated with 2 min activation at 60°C and a 5 min denaturation at 95°C, followed by 40 cycles of 15 s at 95°C and 1 min at 60°C. The expression levels of 18s rRNA were used to normalize the data from the different samples, and the data analysis was performed using the relative quantitation/comparative CT (ΔΔCT) method.

### Phylogenetic Analysis

Amino acid sequences of orthologs of CtA were obtained from NCBI database. Multiple sequence alignments were performed with ClustalX 2.0 (Larkin et al., 2007), and the phylogenetic trees by maximum likelihood was generated by Mega X with 1000 bootstrap replications using Jones-Taylor-Thornton (JTT) model, rates among sites were uniform, and gaps and missing data were completely deleted (Kumar et al., 2018).

### Cloning of the Predicted Cellodextrin Transporter Genes Into *S. cerevisiae*

The predicted cellodextrin transporter genes, *An12g09270* and *An16g06220* (named as *ctA* and *ctB*), from *A. niger* B2 were amplified by using the cDNA as the template, and the primers were listed in Supplementary Table S2. The genes *ctA* and *ctB* were cloned into a pRS426 plasmid, creating plasmids pRS426-ctA and pRS426-ctB (Galazka et al., 2010). *S. cerevisiae* YPH499 (*MAT*a *ura3-52 lys2-801_amber ade2-101_ochre trp1-*Δ*63 his3-*Δ*200 leu2-*Δ*1*) was used as the host for the cellodextrin transport assay (Sikorski and Hieter, 1989). The plasmid pRS425 harboring the *N. crassa* intracellular β-glucosidase gene (*gh1-1*) was transformed into the *S. cerevisiae* YPH499 competent cells by electroporation (1.5k V, 25 μF, 200 Ω, 5 ms), creating the strain YPβG (Galazka et al., 2010). The plasmid pRS426-ctA or pRS426-ctB was then transformed into YPβG in the same way mentioned above, creating the yeast strains YPβG-CtA and YPβG-CtB. The transformants were selected on SM plate containing glucose without leucine or leucine and uracil.

### Fermentation and Cellodextrins Uptake Assay of YPβG-CtA and YPβG-CtB

In the fermentation experiments, the YPβG-CtA strain was first grown aerobically on SM-Leu-Ura medium (50 mL) containing 20 g/L glucose for 24 h. Cells were then harvested by centrifugation and were washed 3 times with sterile water 50 mL. Cell pellets were resuspended in SM-Leu-Ura medium (50 mL) containing cellobiose (20 g/L) and CuSO_4_ (0.2 mM) to a final OD_600_ of 0.5. The mixtures were transferred into serum bottles (100 mL), sealed and incubated at 30°C and 120 rpm. Samples were taken at various time intervals.

In cellodextrins uptake assay, flask (250 mL) containing SM-Leu-Ura medium (50 mL) with CuSO_4_ (0.2 mM) and cellobiose (5 g/L), cellotriose (2.5 g/L), cellotetraose (2.5 g/L), or cellopentaose (2.5 g/L) was inoculated with YPβG-CtA or YPβG-CtB to the final OD_600_ of 0.1. Flasks were incubated at 30°C and 250 rpm. The cell density was monitored by measuring the optical density at 600 nm. An aliquot of 1 mL culture was collected and analyzed by HPLC for measuring the residual cellodextrins.

### Sample Analysis

Concentrations of cellodextrins (cellobiose, cellotriose, cellotetraose, or cellopentaose) and ethanol in the culture samples were analyzed using an UltiMate 3000 HPLC equipped with a refraction index detector and an Aminex HPX-87H (Bio-rad, Hercules, CA, United States). The eluent was H_2_SO_4_ (5 mM) and was eluted at a flow rate of 0.6 mL/min.

## Results

### Exploration of the Cellodextrin Transporters in *A. niger*

The analysis of transcriptome and genome sequencing data of *A. niger* CBS 513.88 showed that it had 86 putative sugar transporters (Pel et al., 2007; de Vries et al., 2017; Peng et al., 2018). The cellodextrin transporter gene candidates were selected based on their protein sequence identity to *N. crassa* CDT-1 or CDT-2 in the *A. niger* CBS 513.88 protein database (Galazka et al., 2010). Seven predicted sugar transporters (An12g09270, An14g01600, An13g03250, An03g05320, An08g09350, An16g06220, and An04g02790) having over 90% coverage and 30% identity to CDT-1 or CDT-2 were selected in this study (Supplementary Table S3). The selected seven putative sugar transporters were further analyzed in Transporter Classification Database (TCDB) (Saier et al., 2006), and the results showed that the gene at locus An12g09270 (36% identity to *N. crassa* CDT-1, *e*-value of 3e-113) and locus An14g01600 (38% identity to *N. crassa* CDT-2, *e*-value of 3e-112) were annotated as MFS lactose permease and MFS hexose transporter, respectively. The others having the best hit to CDT-2 were annotated as sugar transporters (Supplementary Table S3).

To shed more light on the transcription of these 7 predicted cellodextrin transporters’ gene in response to cellulosic biomass, a transcriptional analysis was performed by RT-qPCR for these transporter-coding genes in *A. niger* B2. The changes in transcriptional levels of those predicted cellodextrin transporter genes are shown in Table 1. The gene at locus An12g09270 was highly induced in the sugarcane bagasse medium, resulting in about a 12-fold up-regulation compared to the control. The gene at locus An16g06220 was also induced in the sugarcane bagasse medium, resulting in a two-fold up-regulation compared to the control. However, the other 5 tested genes were not induced by cellulosic biomass efficiently (less than a two-fold increase).

**TABLE 1:** The relative transcription levels of predicted cellodextrin transporter genes in *A. niger* B2 on steam-exploded sugarcane bagasse 0.5%(w/v) compared to growth on glucose reference control.

**Gene**	**2^–ΔΔ^*^*Ct*^***
An12g09270	12.6 ± 0.3
An14g01600	0.35 ± 0.04
An13g03250	0.18 ± 0.05
An03g05320	0.14 ± 0.01
An08g09350	0.57 ± 0.02
An16g06220	1.68 ± 0.1
An04g02790	0.16 ± 0.01

The here identified, putative lactose permease gene (*lac12*, An12g09270) and gene An16g06220 have identity with the *N. crassa* cellobiose transporter *cdt-1* (36% identity, *e*-value of 3e-113) and *cdt-2* (33% identity, *e*-value of 5e-91), respectively. These two genes were selected for further functional analysis, and here now were named *ctA* and *ctB*, respectively.

### Investigation on the Ability of CtA and CtB to Transport Cellobiose

To evaluate the ability of CtA and CtB to transport cellobiose, both genes were amplified from *A. niger* B2 and were cloned into *S. cerevisiae* YPβG that harbored the *N. crassa*-glucosidase gene *gh1-1* (NCU00130). The expression of a functional cellodextrin transporter will enable the YPβG strain to grow on the medium with cellobiose as the sole carbon source, which has been demonstrated in the previous work that we participated in Galazka et al. (2010). Herein, the strain YPβG-CtA and YPβG-CtB, harboring the predicted cellodextrin transporter gene *ctA* and *ctB*, respectively, were constructed.

**FIGURE 1:**
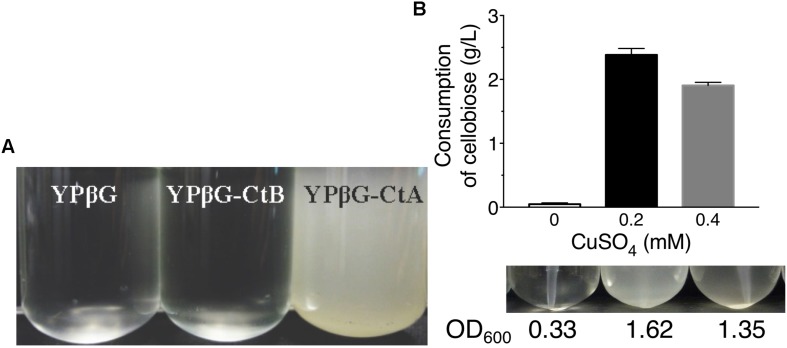
The growth of yeast strains in the media with cellobiose as the sole carbon source. **(A)** Growths of yeast strain YPβG, YPβG-CtA, and YPβG-CtB in SM-Leu or SM-Leu-Ura media with cellobiose as the sole carbon source after 2 days. **(B)** The cellobiose utilization and cell growth of YPβG-CtA strain in the media containing a different concentration of copper.

**FIGURE 2:**
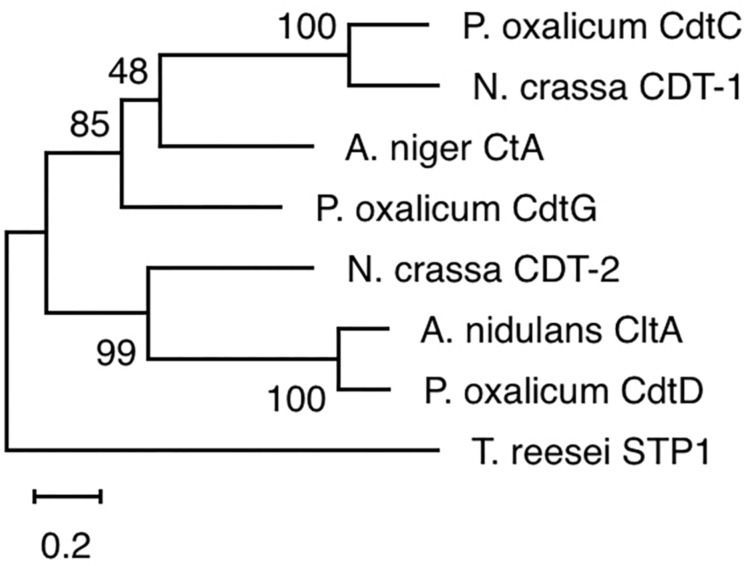
Phylogenetic analysis of CtA and other cellobiose/cellodextrin transporters. Sequence alignments were performed with ClustalX 2.0, and the maximum likelihood tree was generated with Mega X. Numbers on the tree branches represent the bootstrap support calculated per 1000 bootstrap replicates. CdtC, *Penicillium oxalicum* cellodextrin transporter (GenBank No. EPS25673.1); CDT-1, *Neurospora crassa* cellodextrin transporter (GenBank No. XP_963801.1); CdtG, *Penicillium oxalicum* cellodextrin transporter (GenBank No. EPS34431.1); CDT-2, *Neurospora crassa* cellodextrin transporter (GenBank No. XP_963873.1); CltA, *Aspergillus nidulans* cellobiose transporter (GenBank No. AN8347); CdtD, *Penicillium oxalicum* cellodextrin transporter (GenBank No. EPS25817.1); STP1, *Trichoderma reesei* cellodextrin transporter (GenBank No. ETS01554.1).

The yeast strains YPβG, YPβG-CtA and YPβG-CtB were inoculated into the SM-Leu or SM-Leu-Ura media with cellobiose as the sole carbon source to test the transportability of cellobiose (Figure 1A). The results showed that YPβG strain only harboring the β-glucosidase gene could not grow in the medium, while YPβG-CtA strain harboring the *ctA* gene and β-glucosidase gene could grow on cellobiose. Therefore, CtA’s function as a cellobiose transporter was confirmed. However, YPβG-CtB strain harboring the *ctB* gene and β-glucosidase gene could not grow in the medium, indicating that the CtB dose not have the ability to transport cellobiose into the cells. Herein, the sequence of *ctA* was deposited in GenBank under the accession number of MH648002. The cellodextrin transporter CtA in *A. niger* shares 36% identity with CDT-1 (Galazka et al., 2010), but only 29% identity with the cellobiose transporter CltA in *A. nidulans* (dos Reis et al., 2016). Phylogenetic analysis further revealed that CtA was closely related to the well characterized cellodextrin transporter CDT-1 from *N. crassa* and CdtC from *P. oxalicum*, but did not group with CltA from *A. nidulans* (Figure 2).

Since copper is the inducer for Cup1 promoter used in pRS426 plasmid expressing transporter genes, the copper concentration in the culture was investigated for efficient cellobiose utilization (Mascorro-Gallardo et al., 1996). As shown in Figure 1B, no obvious cell growth of YPβG-CtA and cellobiose consumption in the culture was observed when no copper was added in the media. When the copper concentration in the medium was 0.2 mM, about 50% cellobiose was consumed in 2 days, and the cell density in terms of OD_600_ was up to 1.6. However, when the copper concentration in the medium was increased to 0.4 mM, the strain utilized less cellobiose and achieved a lower cell density than those of 0.2 mM copper, indicating that 0.2 mM copper was optimal for CtA expression.

**FIGURE 3:**
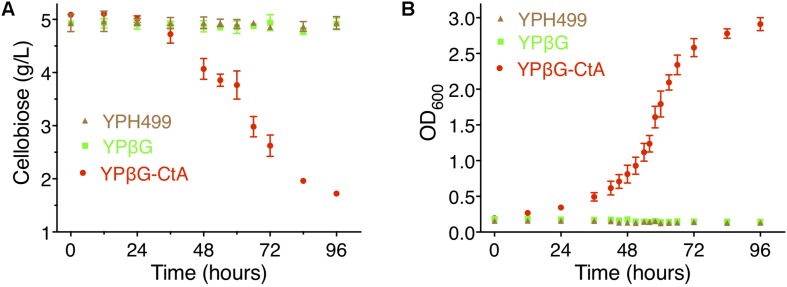
The cellobiose mediated growth of *S. cerevisiae* strain YPH499, YPβG, and YPβG-CtA. **(A)** The time course of cellobiose concentration of the YPH499, YPβG, and YPβG-CtA strains in the cultures with 0.5% cellobiose as the sole carbon source. **(B)** Growth curve of *S. cerevisiae* strains. The YPβG-CtA strain expressing transporter *ctA* and *bgl* consumed cellobiose, while the YPH499 and YPβG strains could not utilize cellobiose.

To further investigate the efficiency of CtA to transport cellobiose, the yeast strain YPH499, YPβG, and YPβG-CtA were cultured in the SM media, SM-Leu media or SM-Leu-Ura media with cellobiose as the sole carbon source, and the cell growth and concentration of cellobiose in the cultures were analyzed. It was shown that YPβG-CtA consumed about 3.5 g/L cellobiose in 4 days (Figure 3A). The cellobiose consumption rate during the exponential growth was 57.5 mg/L/h (Figure 3B). Cell density reached an OD_600_ of 3.0. However, no cellobiose consumption and cell growth with YPH499 and YPβG strains were observed.

### Investigation on the Ability of CtA and CtB to Transport Cellodextrins

**FIGURE 4:**
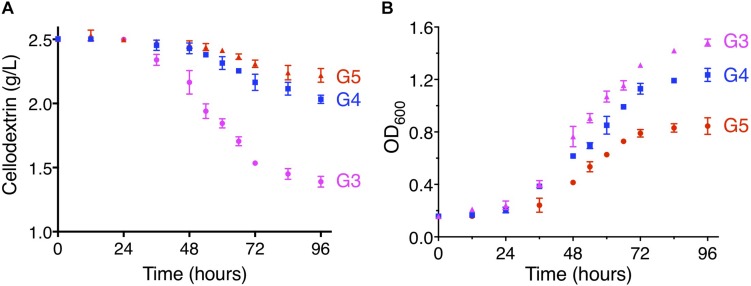
The consumption of cellodextrins **(A)** and cell growth **(B)** of the YPβG-CtA strain. The yeast strain was cultured in the SM-Leu-Ura medium containing 0.25% (w/v) of cellotriose (G3), cellotetraose (G4), or cellopentaose (G5) as the sole carbon source at 30°C and 250 rpm.

To further examine the ability to transport cellodextrins of CtA and CtB, the YPβG-CtA and YPβG-CtB were cultured in the SM-Leu-Ura media with cellotriose, cellotetraose, or cellopentaose as the sole carbon source. The YPβG-CtB strain could not grow in the medium containing cellotriose, cellotetraose, or cellopentaose and did not consume those cellodextrins, indicating that the CtB is not a cellodextrin transporter. The YPβG-CtA strain consumed 1.08 ± 0.10 g/L cellotriose, 0.5 ± 0.08 g/L cellotetraose, and 0.24 ± 0.09 g/L cellopentaose in 4 days (Figure 4). The OD_600_ reached 3.0, 1.5, 1.2, and 0.8 after 4 days culture when cellobiose, cellotriose, cellotetraose, and cellopentaose were used as the carbon source. The corresponding substrate consumption rates in the exponential growth phase are 57.5, 18.1, 8.3, and 4.1 mg/L/h (Figures 3, 4). Thus, CtA was able to transport all the tested G2-G5 cellodextrins into the cells. However, CtA had lower activity in the transportation of cellotriose, cellotetraose, and cellopentaose than that of cellobiose.

### Ethanol Production of YPβG-CtA on Cellobiose

**FIGURE 5:**
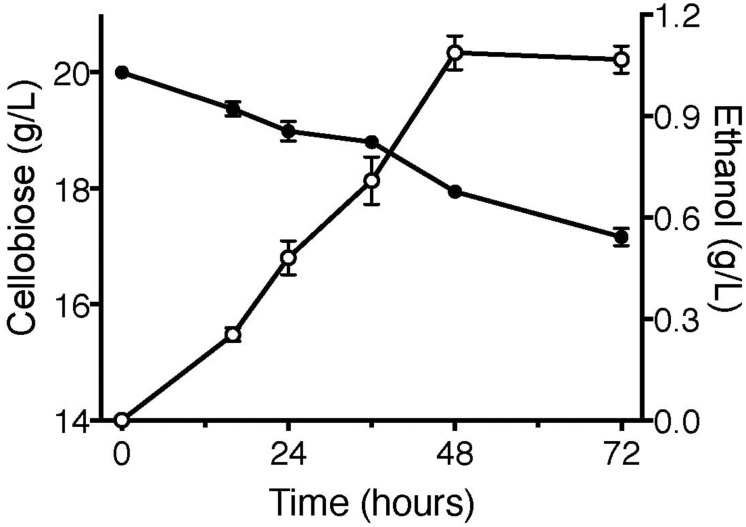
The anaerobic fermentation of YPβG-CtA on cellobiose.

Cellodextrin transporters enable the non-cellobiose fermenting *S. cerevisiae* and *E. coli* to uptake cellodextrins, facilitating the engineered microorganisms to produce biofuels (Ha et al., 2011; Vinuselvi and Lee, 2011; Bae et al., 2014; Fan et al., 2016). To further confirm the function of CtA, the fermentation of YPβG-CtA on cellobiose (20 g/L) was conducted anaerobically, and the cellobiose consumption and ethanol production were analyzed (Figure 5). The YPβG-CtA utilized 2.2 g/L cellobiose in 2 days and yielded 1.1 g/L ethanol, which is 83.4% of the theoretical value. However, extending the fermentation time did not yield more ethanol with about 3 g/L cellobiose consumed after 3 days. The anaerobic fermentation of YPβG-CtA strain on cellobiose resulted in a slight lower ethanol production efficiency than the engineered strain harboring CDT-1 (Galazka et al., 2010). It is expected that the ethanol production efficiency of YPβG-CtA can be further improved by optimizing the fermentation conditions.

## Discussion

In this study, two (An12g09270 and An16g06220) of the seven predicted sugar transporters genes were up-regulated when *A. niger* was grown on steam-exploded sugarcane bagasse, and the CtA (An12g09270) was confirmed to be a novel cellodextrin transporter. The phylogenetic analysis of the orthologs of CtA showed that it did not group with cellobiose transporter CltA from *A. nidulans* (Figure 2), which indicated CtA is a novel cellodextrin transporter in *Aspergillus* species. The further characterization of CtA showed that it was able to transport all the tested G2–G5 cellodextrins into the cells, while the transportation activities on the different substrates of CtA decreased slightly with increasing chain length (Figures 3, 4). The ability of CtA to transport G2–G5 cellodextrins, combined with the fact provided by de Vries et al. (2017) that none of the 12 tested sugars (including 8 monosaccharides, turanose, trehalose, maltose, and melitose) could be transported by An12g09270 (CtA), may suggest that CtA is a specific transporter for cellodextrin transportation in *A. niger*.

**FIGURE 6:**
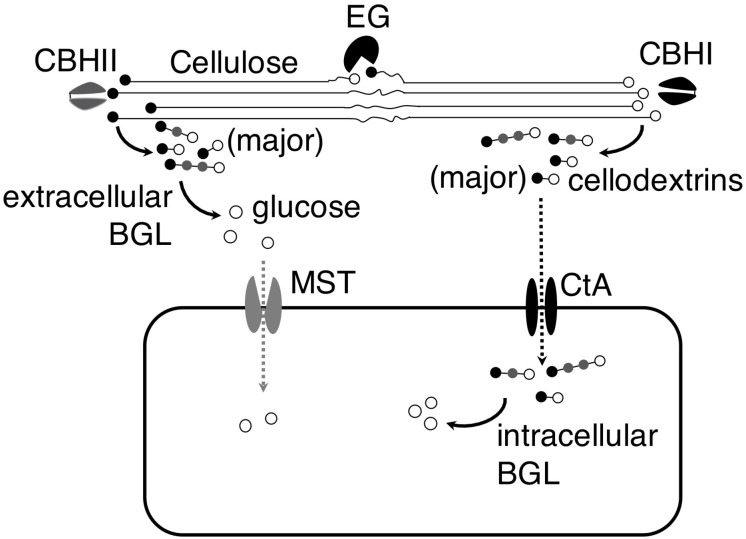
Model of cellulose utilization in *A. niger* by both complete and partial hydrolysis strategies. In both strategies, cellulose is first transformed into cellobiose and cellodextrins by endoglucanase (EG) and exo-cellobiohydrolases (CBHI and CBHII). In the complete hydrolysis strategy, extracellular β-glucosidase (BGL) hydrolyzes the cellobiose and cellodextrins into glucose and then it is transferred into the cells by monosaccharide transporter (MST). However, the cellobiose and cellodextrins are transferred into the cells by cellodextrin transporter (CtA) and then they are hydrolyzed into glucose by intracellular BGL in the partial hydrolysis strategy.

The identification of the cellodextrin transporter in *A. niger* led us to hypothesize that *A. niger* could import partial hydrolysis products, cellodextrins, and utilize them intracellularly, in addition to completely hydrolyzing cellodextrins to glucose and uptaking glucose. The analysis of *A. niger* CBS 513.88 genome showed that the fungus contains 5 putative BGLs without signal peptide (Supplementary Table S4; Pel et al., 2007; Petersen et al., 2011), and two (An03g03740 and An17g00520) of the 5 putative intracellular BGLs were showed as cellulosic inducible genes (de Souza et al., 2011), indicating their relevance to cellodextrins’ intracellular utilization. The existence of the putative intracellular BGLs in *A. niger* genome corroborated our hypothesis. The work provided a new understanding of the strategies of cellulose utilization in *A. niger*. When *A. niger* grows on cellulose, the fungus secrets cellulases to degrade cellulose into cellobiose and cellodextrins, and then cellobiose and cellodextrins are either subject to complete hydrolysis by extracellular BGLs into glucose followed by transportation into the cells by monosaccharide transporter (MST), or directly transported into the cells through cellodextrin transporter CtA followed by degradation by intracellular BGLs (Figure 6). Since *A. niger* secrets an enormous amount of BGLs, the complete hydrolysis strategy plays a major role in its cellulose utilization.

The expression of *ctA* in the *S. cerevisiae* harboring a *N. crassa* β-glucosidase gene *gh1-1* (YPβG-CtA) enabled the strain to use cellobiose efficiently and produced ethanol under the anaerobic fermentation. The YPβG-CtA strain had the cell growth rate of 0.0830 ± 0.0113 h^–1^ (Figure 3B), which was faster than the engineered yeast strain expressing the widely used *N. crassa cdt-1* or *cdt-2* and the same β-glucosidase gene *gh1-1*, which had the cell growth rate of 0.0341 ± 0.0010 h^–1^ and 0.0131 ± 0.0008 h^–1^, respectively (Galazka et al., 2010). It showed that CtA was a more efficient transporter, which enabled the engineered yeast strain to uptake cellobiose faster than *N. crassa* CDT-1 or CDT-2. Together with the ability to transport G2–G5 cellodextrins (Figure 4), CtA is an attractive choice of the cellodextrin transporter for engineering cellobiose or cellodextrin utilization microorganism.

## Conclusion

In this study, the cellodextrin transporter CtA in *A. niger* was identified and characterized. Heterologously expressing the identified cellodextrin transporter CtA gene together with a β-glucosidase gene enabled the engineered *S. cerevisiae* strain to grow on cellobiose and cellodextrins up to chain length five. The engineered strain expressing CtA grew faster in cellobiose medium than the yeast strain harboring the widely used *N. crassa* cellobiose transporter CDT-1, which made it a strong transporter candidate for constructing engineered cellodextrin-utilizing microorganisms.

## Data Availability Statement

The datasets supporting the conclusions of this article are included within the article (and its additional file). The sequence of CtA has been deposited in the GenBank under the accession number of MH648002.

## Author Contributions

HC, CT, and HL designed the study. QZ, SC, and JZ carried out the experiments. HC, HL, QZ, and SC analyzed the data. HC, HL, and ZF wrote and edited the manuscript. All authors read and approved the final manuscript.

## Conflict of Interest

The authors declare that the research was conducted in the absence of any commercial or financial relationships that could be construed as a potential conflict of interest.
