# Supplementary material for: Identification and Characterization of a Cellodextrin Transporter in Aspergillus niger
Source: Front Microbiol. 2020 Feb 7;11:145. doi: 10.3389/fmicb.2020.00145 (PMC7020610; doi:10.3389/fmicb.2020.00145)
Supplement: Supplementary file 1 [file Data_Sheet_1.pdf]

Supporting information

Identification and Characterization of a Cellodextrin Transporter in *Aspergillus niger*

Hui Lin[1], Jun Zhao[1], Qingqing Zhang[1], Shixiu Cui[1], Zhiliang Fan[3], Hongge Chen[1*], Chaoguang Tian[2*]

[1] College of Life Sciences, Henan Agricultural University, 95 Wenhua Road, Zhengzhou 450002, China

[2] Tianjin Institute of Industrial Biotechnology, Chinese Academy of Sciences, 32 West 7th Avenue, Tianjin Airport Economic Area, Tianjin 300308, China

[3] Biological and Agricultural Engineering Department, University of California, Davis, One Shields Avenue, Davis, CA 95616

[*]Corresponding author:

HC: honggeyz@henau.edu.cn

CT: tian_cg@tib.cas.cn

Table S1. Oligonucleotides were used in the RT-qPCR.

	Oligonucleotides (5’ → 3’)
An03g03740-F	ATGGGTTCAGCAACAGCTTC
An03g03740-R	TTATTTCTTCTCGATATACTGGCTG
An03g05330-F	ATGCCTTGCGTACAGGCC
An03g05330-R	TCAGAGCCCCGACCAG
An06g02040-F	ATGCCTCATGAGGAAAGAGTCTC
An06g02040-R	CTACTCTAGTGTTCCCTGCCTGG
An15g01890-F	ATGATGGTTTGGGCGTTTTT
An15g01890-R	TCAGACCAGGACACACTGTTG
An17g00520-F	ATGGCACGCGTGGACTTT
An17g00520-R	CTACAACCCAACCCAATACCTCG
An12g09270-F	CTGGCTCATCTCACAAGACAAG
An12g09270-R	TAGACCGATACCGCACTTCC
An14g01600-F	CAGTGTGGATGGGCGAATTAG
An14g01600-R	CTTGGAAGACGGTGGGAATG
An13g03250-F	CAGCCTCTTGGGAACCATAGT
An13g03250-R	CCGACGCACATTGGATAGTTG
An03g05320-F	TCCGAGATCAGTTCCACCAT
An03g05320-R	TGAGCGACAGGTAATACGAGAC
An08g09350-F	CTGCTCGTTCCGTTACTAT
An08g09350-R	GGTTAGGCGTCTTCCAAT
An16g06220-F	ACTCTCAGTCTTGTCTTGTT
An16g06220-R	TAAGCCAGAAGCAATCCTAT
An04g02790-F	CGTGCCGTGATTGGTTGT
An04g02790-R	GCCGTCATACGCTTGTGTT
18S-F	ACTCACCAGGTCCAGACAAAATAAG
18S-R	AAGCAGACAAATCACTCCACCA

Table S2. Oligonucleotides for the amplification of *bgl*s and cellodextrin transporter genes.

	Oligonucleotides (5’ → 3’)
bgl1-F	CCGAATTCCACGTGCATGGGTTCAGCAACAGCTTCA
bgl1-R	TTAATTCCACGCGGCCGCTTTCTTCTCGATATACTGGCTGAAAATC
bgl2-F	CCGAGATACACGTGCATGGCACGCGTGGACTTT
bgl2-R	TTAATTCCACGCGGCCGCCAACCCAACCCAATACCTCG
AnCtA-F	CCGGAATTCAAAAATGGCTGAGAAAACCGCCACC
AnCtA-R	CCCAAGCTTCTTCATCTCCTCGATCTCCGTAAGATCC
AnCtB-F	CCGGAATTCAAAAATGGACTTGAAGATGGCCGAGAA
AnCtB-R	ATAAGCTTGTCAGTCGCCCTGGTTGACGT

Table S3. Seven predicted cellodextrin transporters in *A. niger* CBS 513.88 with over 30% identity to CDT-1 or CDT-2 from *N. crassa*.

Gene locus	Best-Hit	Identity (%)	E-value	Annotation
An12g09270	cdt-1	36	3e-113	MFS lactose permease
An14g01600	cdt-2	38	3e-112	MFS hexose transporter
An13g03250	cdt-2	37	6e-105	Sugar transporter
An03g05320	cdt-2	36	3e-104	Sugar transporter
An08g09350	cdt-2	36	1e-101	Sugar transporter
An16g06220	cdt-2	33	5e-91	Sugar transporter
An04g02790	cdt-2	32	3e-89	Sugar transporter

Table S4. Predicted intracellular β-glucosidases in *Aspergillus niger* CBS 513.88.

Gene locus	CAZy Module(s)	Signal peptide	Inducible [a]
**An03g03740**	**GH 1**	**No**	+
An03g05330	GH 3	No	-
An06g02040	GH 3	No	-
An15g01890	GH 3	No	-
**An17g00520**	**GH 3**	**No**	+

[a] "+" means sugarcane bagasse inducible gene; "_" means sugarcane bagasse noninducible gene.

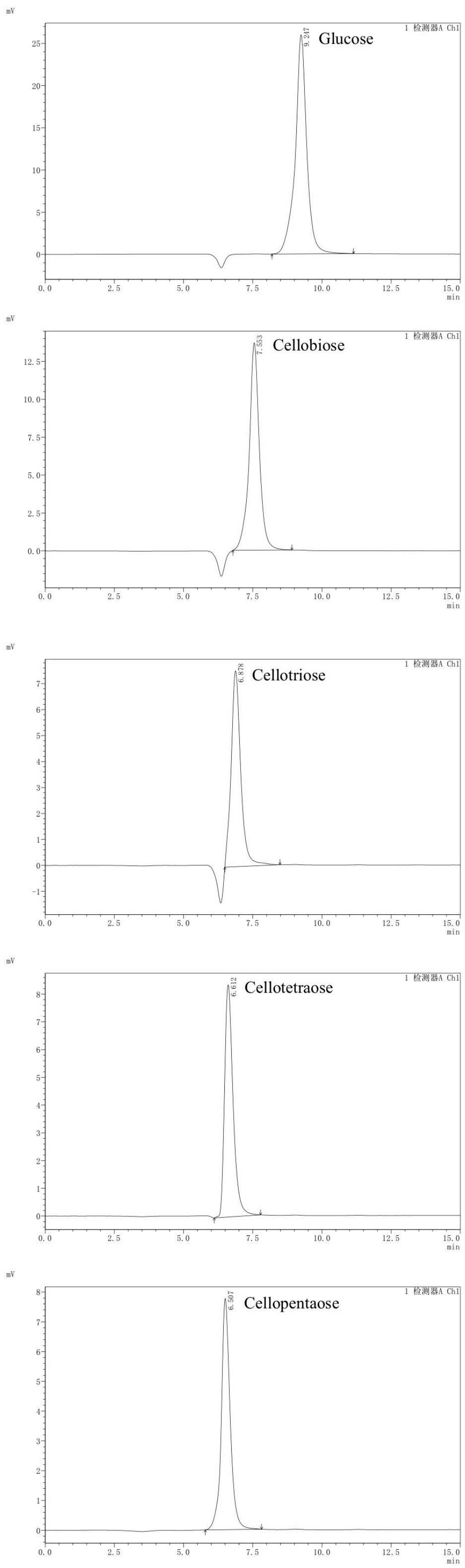

Figure S1. The HPLC profiles of glucose, cellobiose, cellotriose, cellotetraose, and cellopentaose.
